# Supplementary material for: TRPC5 Channel Inhibition Protects Podocytes in Puromycin-Aminonucleoside Induced Nephrosis Models
Source: Front Med (Lausanne). 2021 Sep 21;8:721865. doi: 10.3389/fmed.2021.721865 (PMC8490698; doi:10.3389/fmed.2021.721865)
Supplement: Supplementary Methods — Total mRNA was extracted from 5 × 10^6^ hiPSC and iPodo cells with TRIzol reagent according to the official protocol (ThermoFisher Scientific, USA). The cDNA was synthesized by ReverTra Ace kit (Toyobo, Japan). qPCR was then performed using a SYBR Green qPCR Kit (ThermoFisher Scientific, US) with the CFX Real-Time PCR system (Bio-Rad, USA). GAPDH (Forward 5′-GTCTCCTCTGACTTCAACAGCG-3′, Reverse 5′-ACCACCCTGTTGCTGTAGCCAA-3′); SYNPO (Forward 5′-TCCTTCATGTTGCTGCCGAT-3′, Reverse 5′-AGATCCTTCTCCGTGAGGCT-3′); NPHS2 (Forward 5′-ACCAAATCCTCCGGCTTAGG-3′, Reverse 5′-CAACCTTTACGCAGAACCAGA-3′); NPHS1 (Forward 5′-GTCTGCACTGTCGATGCCAATC-3′, Reverse 5′-CCAGTTTGGCATGGTGAATCCG-3′); WT1 (Forward 5′-CGAGAGCGATAACCACACAACG-3′, Reverse 5′-GTCTCAGATGCCGACCGTACAA-3′). [file Data_Sheet_1.docx]

**Supplementary Methods |** Total mRNA was extracted from 5 × 106 hiPSC and iPodo cells with TRIzol reagent according to the official protocol (Thermo Fisher Scientific, USA). The cDNA was synthesized by ReverTra Ace kit (Toyobo, Japan). qPCR was then performed using a SYBR Green qPCR Kit (ThermoFisher Scientific, US) with the CFX Real-Time PCR system (Bio-Rad, USA). *GAPDH* (Forward 5′-GTCTCCTCTGACTTCAACAGCG-3′, Reverse 5′-ACCACCCTGTTGCTGTAGCCAA-3′); *SYNPO* (Forward 5′-TCCTTCATGTTGCTGCCGAT-3′, Reverse 5′-AGATCCTTCTCCGTGAGGCT-3′); *NPHS2* (Forward 5′-ACCAAATCCTCCGGCTTAGG-3′, Reverse 5′-CAACCTTTACGCAGAACCAGA-3′); *NPHS1* (Forward 5′-GTCTGCACTGTCGATGCCAATC-3′, Reverse 5′-CCAGTTTGGCATGGTGAATCCG-3′); *WT1* (Forward 5′-CGAGAGCGATAACCACACAACG-3′, Reverse 5′-GTCTCAGATGCCGACCGTACAA-3′).


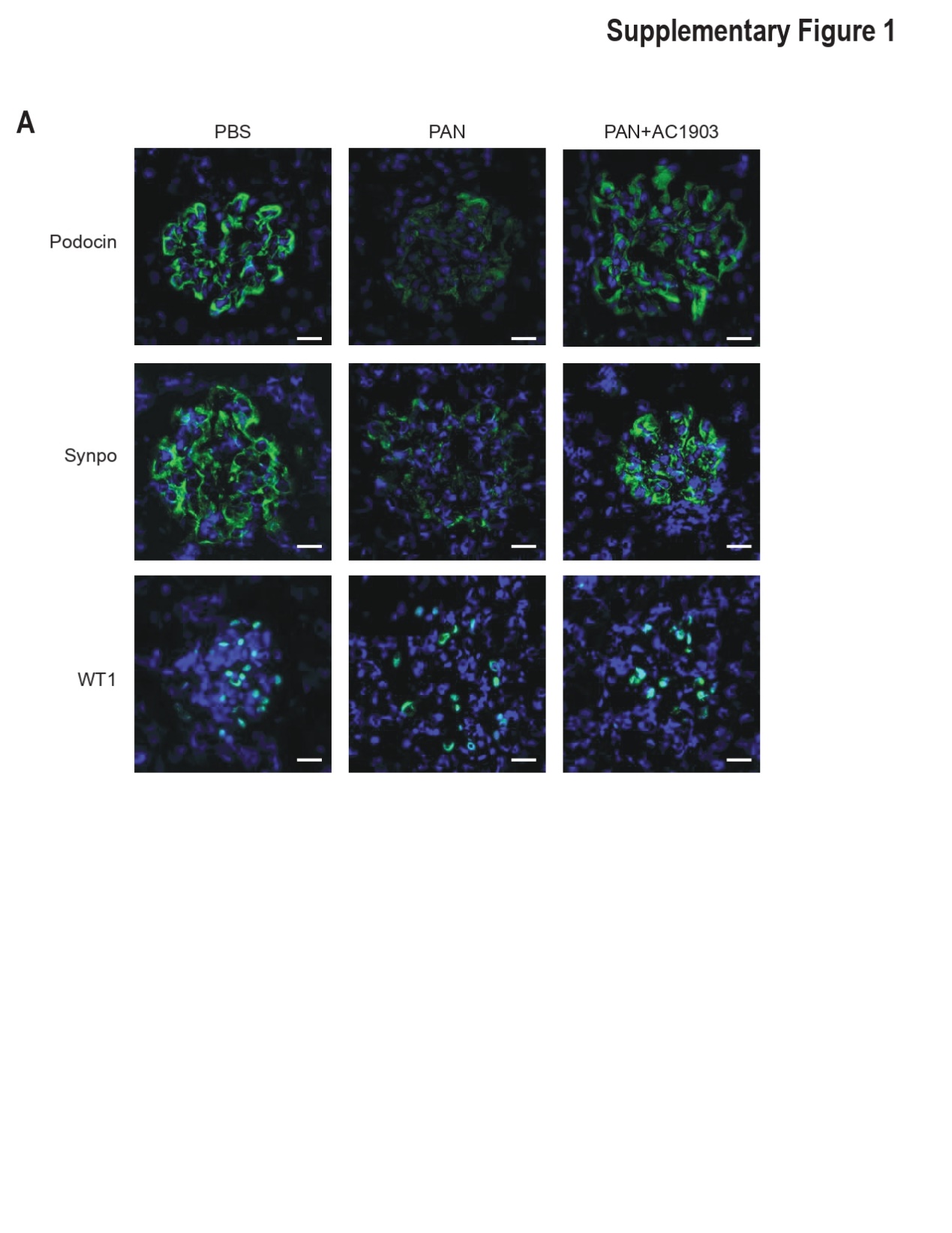


**Supplementary Figure 1 |** Inhibition of TRPC5 channel activity protects podocytes in PAN rats. **(A)** Representative immunostaining images of podocyte cytoskeletal and marker proteins podocin, synaptopodin, and WT1 from PBS, PAN and PAN + AC1903 treated rats on day 7. Scale bar 20 µm.


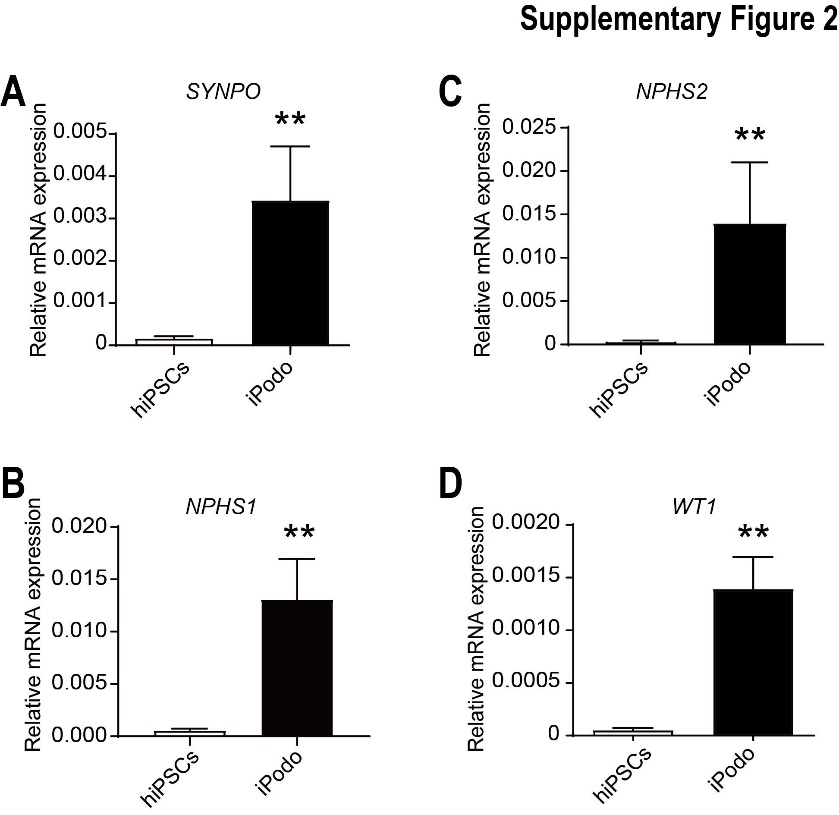


**Supplementary Figure 2 |** Expression levels of *SYNPO*, *NPHS1*, *NPHS2*, and *WT1* in human iPSC-derived podocytes. **(A–D)** qPCR results of podocyte markers SYNPO **(A)**, NPHS1 **(B)**, NPHS2 **(C)**, and WT1 **(D)** from hiPSC and iPodo. hiPSC *n* = 3, iPodo *n* = 3. ∗∗*p* < 0.01 vs. hiPSC.


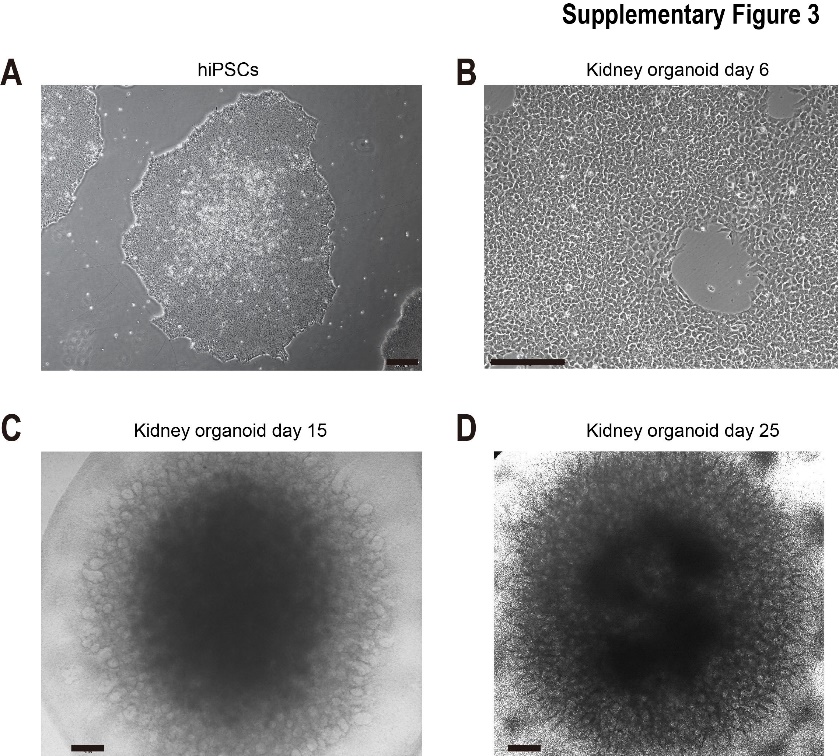


**Supplementary Figure 3 |** Bright-field images of human iPSCs and kidney organoids. **(A)** Bright-field images of human iPSCs (**A**, scale bar 200 µm), kidney organoid at day 6 **(B)**, day 15 **(C)**, and day 25 **(D)**. Scale bars **(B–D)** 50 µm.


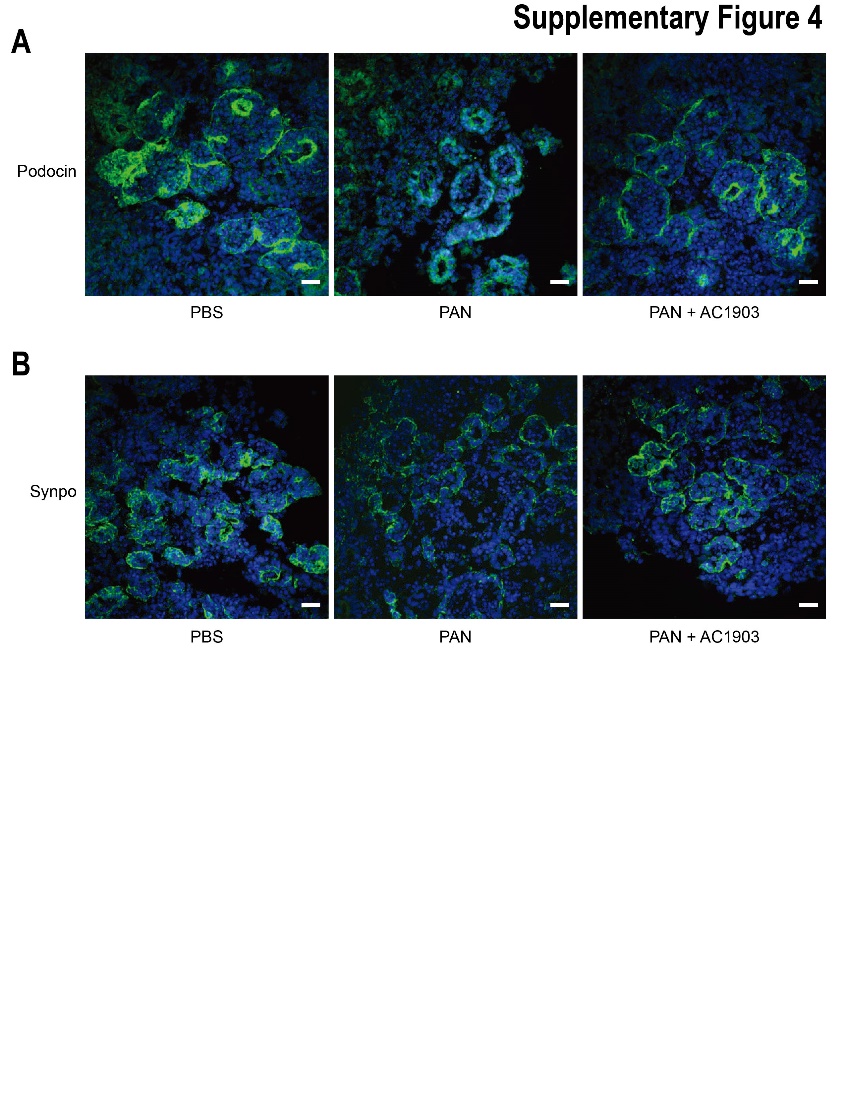


**Supplementary Figure 4 |** Inhibition of TRPC5 activity protects podocin and synaptopodin in human kidney organoids treated with PAN. **(A, B)** Immunostaining for podocin **(A)** and synaptopodin **(B)** in PBS, PAN and PAN + AC1903 treated kidney organoids. Scale bar 20 µm.
